# Alkaline ceramidase 1 is essential for mammalian skin homeostasis and regulating whole‐body energy expenditure

**DOI:** 10.1002/path.4737

**Published:** 2016-05-30

**Authors:** Kifayathullah Liakath‐Ali, Valerie E Vancollie, Christopher J Lelliott, Anneliese O Speak, David Lafont, Hayley J Protheroe, Camilla Ingvorsen, Antonella Galli, Angela Green, Diane Gleeson, Ed Ryder, Leanne Glover, Gema Vizcay‐Barrena, Natasha A Karp, Mark J Arends, Thomas Brenn, Sarah Spiegel, David J Adams, Fiona M Watt, Louise van der Weyden

**Affiliations:** ^1^ Centre for Stem Cells and Regenerative MedicineKing's College LondonUK; ^2^ Department of BiochemistryUniversity of CambridgeUK; ^3^ Wellcome Trust Sanger InstituteHinxtonCambridgeUK; ^4^ University of Cambridge Metabolic Research LaboratoriesWellcome Trust–MRC Institute of Metabolic Science, Addenbrooke's HospitalCambridgeUK; ^5^ University of Edinburgh Division of Pathology, Edinburgh Cancer Research CentreInstitute of Genetics and Molecular Medicine, Western General HospitalEdinburghUK; ^6^ NHS Lothian University Hospitals Trust and University of EdinburghDepartment of Pathology, Western General HospitalEdinburghUK; ^7^ Centre for Ultrastructural ImagingKing's College LondonGuy's CampusLondonUK; ^8^ Department of Biochemistry and Molecular BiologyVirginia Commonwealth University School of MedicineRichmondVAUSA

**Keywords:** skin homeostasis, ceramidase, sebaceous glands, energy homeostasis

## Abstract

The epidermis is the outermost layer of skin that acts as a barrier to protect the body from the external environment and to control water and heat loss. This barrier function is established through the multistage differentiation of keratinocytes and the presence of bioactive sphingolipids such as ceramides, the levels of which are tightly regulated by a balance of ceramide synthase and ceramidase activities. Here we reveal the essential role of alkaline ceramidase 1 (Acer1) in the skin. Acer1‐deficient (Acer1^−/−^) mice showed elevated levels of ceramide in the skin, aberrant hair shaft cuticle formation and cyclic alopecia. We demonstrate that Acer1 is specifically expressed in differentiated interfollicular epidermis, infundibulum and sebaceous glands and consequently Acer1 ^−/−^ mice have significant alterations in infundibulum and sebaceous gland architecture. Acer1^−/−^ skin also shows perturbed hair follicle stem cell compartments. These alterations result in Acer1^−/−^ mice showing increased transepidermal water loss and a hypermetabolism phenotype with associated reduction of fat content with age. We conclude that Acer1 is indispensable for mammalian skin homeostasis and whole‐body energy homeostasis. © 2016 The Authors. *The Journal of Pathology* published by John Wiley & Sons Ltd on behalf of Pathological Society of Great Britain and Ireland.

## Introduction

The skin functions as a barrier that protects the body from the external environment and also controls water and heat loss. Barrier function is established through the multistage differentiation of keratinocytes in the epidermis, in which keratinocytes differentiate from proliferative cells in the basal layer into cornified cells in the stratum corneum. Ceramide is the main sphingolipid component of the stratum corneum, which is comprised of terminally differentiated corneocytes with characteristic multilamellar membrane unit structures that mediate barrier function 1. Altered expression of ceramides has been identified in human skin conditions such as psoriasis and atopic dermatitis 2, 3.

The levels of ceramide in the skin are tightly regulated by the activities of ceramide synthases (CerSs), which synthesize ceramides, and ceramidases, which hydrolyse ceramides. To date, seven ceramidases have been identified, categorized by their pH optima: acid ceramidase (ASAH1), neutral ceramidase (ASAH2, ASAH2B, ASAH2C) and alkaline ceramidases 1–3 (ACER1–3).

ACER1 is highly expressed in the epidermis 4, 5 and it has been shown that upregulation of ACER1 facilitates differentiation of cultured human keratinocytes 5, although the role of ACER1 in the skin in vivo is unknown. To study the role of Acer1 in skin homeostasis, we used Acer1‐deficient mice. We characterized the abnormal hair follicle formation and cycling, epidermal hyperplasia and sebaceous gland and infundibulum abnormalities that are seen in these mice, along with the associated increased epidermal water loss and hypermetabolism, all of which highlight the key role of ceramides in maintaining the normal barrier and thermoregulatory functions of the skin.

## Materials and methods

### Mice

Acer1^tm1a(EUCOMM)Wtsi^ mice were generated at the Sanger Institute as part of the European Conditional Mouse Mutagenesis Programme and Knockout Mouse Project (EUCOMM/KOMP) projects and Sanger Institute Mouse Pipelines. Mice were generated from ES cell clone EPD0377_3_B06 (available from EMMA resource: https://www.infrafrontier.eu/search?keyword=EM:08275) and backcrossed to C57BL/6 N females, with genotyping carried out as described previously 6 (for more details, see supplementary material). The care and use of all mice in this study was in accordance with UK Home Office regulations and were approved by the Wellcome Trust Sanger Institute and King's College London Animal Welfare and Ethical Review Bodies (for information on housing and husbandry conditions, see supplementary material).

### Histology, whole‐mount staining and imaging

Skin samples were fixed with 10% neutral buffered formalin overnight before paraffin embedding. The tissues were sectioned and stained with haematoxylin and eosin (H&E) by conventional methods (for more information on the antibodies, see supplementary material). Images of H&E‐stained sections were acquired using a Hamamatsu slide scanner and analysed using NanoZoomer software (Hamamatsu). For lacZ staining, a β‐galactosidase reporter gene staining kit (Sigma) was used, following the manufacturer's protocol (for information on the procedure used for oil red O staining of tail epidermal sheets, see supplementary material).

### In vivo phenotyping

Mice undergoing primary phenotyping, including protocols for an initial dysmorphology assessment at 9 weeks and indirect calorimetry at 12 weeks (males only), were studied using a modified version of the Sanger Institute Mouse Pipelines, detailed previously 7, using Mouse Breeders Diet instead of a high‐fat diet. Indirect calorimetry was carried out under normal facility conditions (21–23 °C). Based on the primary screen data, whole‐body morphology, including examination of the hair, skin and vibrissae, was performed using a standardized checklist of 104 parameters and, where appropriate, images were captured at various ages using a Sony DSC‐HX7V. Further information about these parameters can be found at https://www.mousephenotype.org/impress/protocol/185/15 and in Table S3 (see supplementary material). An assessment of hair follicle cycling was performed at age 40–43 days. A small patch of fur on the lower dorsal surface was shaved to reveal the skin, whose colour was then assessed: black skin indicated anagen phase, while other shades were non‐anagen phases of the hair growth cycle. Basal transepidermal water loss (TEWL) was assessed on the dorsal skin of mice at age 19 weeks, using a TEWAmeter (Courage and Khazaka, TM210). Measurements were recorded for 15–20 s when TEWL readings were stabilized, at approximately 30 s after the probe collar was placed on the dorsal skin. Data are shown as mean ± standard error (SE). Unpaired Student's t‐test was used to determine statistical significance.

### Embryo whole‐mount dye‐penetration assay

Embryonic day 16.6–18.5 (E16.5–18.5) embryos were obtained from Acer1^+/−^ intercross timed matings, with the mid‐point of the mating window designated as gestational E0.5. The epidermal permeability assay was performed as described previously 8, with minor modifications detailed in supplementary material.

### Electron microscopy

See supplementary material, for information on the procedures used for scanning electron microscopy (SEM) and transmission electron microscopy (TEM) analyses of hair and skin samples.

### Analysis of sphingolipids by liquid chromatography–electrospray ionization–tandem mass spectrometry (LC–ESI–MS/MS)

Internal standards (0.5 nm each; Avanti Polar Lipids, Alabaster, AL, USA) were added to samples, lipids were extracted from dorsal skin and tail skin (separated into the dermis and epidermis) of young (9 weeks) and old (32 weeks) Acer1^+/+^ and Acer1^−/−^ male mice, and sphingolipids were quantified by LC–ESI–MS/MS (4000 QTRAP, AB Sciex, Framingham, MA, USA), as described previously 9.

### Adipose tissue analysis

White adipose tissue (WAT) depots (inguinal and epididymal) and intrascapular brown adipose tissue (iBAT) were isolated from young (9 week‐old) or old (range 31–34, mean 32 weeks) Acer1 ^+/+^ and Acer1 ^−/−^ male mice (n = 5/genotype) and weighed for calculation of total UCP1 content per iBAT depot (for more information on UCP1 determination, see supplementary material). For adipose tissue histopathology, depots were excised from 18 week‐old male Acer1 ^+/+^ and Acer1 ^−/−^ mice (n = 5/genotype) and fixed in 10% neutral buffered formalin (n = 5/genotype) or snap‐frozen in liquid N_2_ (n = 3/genotype). For histological analysis, H&E‐stained sections were examined independently by two pathologists. For adipose tissue Acer1 gene expression, RT–qPCR was performed, as detailed in Supplementary materials and methods (see supplementary material), using tail skin as a positive control for expression.

### Statistics

Data analysis for indirect calorimetry was performed in R, using ANCOVA analysis and correcting for: (a) body weight, to calculate energy expenditure; and (b) changes in body weight, to calculate food intake. Primary dysmorphological observations at 9 weeks were analysed compared to a global reference range combining all wild‐type mice run in the pipeline, as described in 7. Secondary dysmorphology parameters at 6 and 10 weeks were analysed by Biased Reduction Logistic Regression in R, using the package Phenstat 2.0.1 10, comparing to local wild‐type controls. All other analysis was performed using statistical tests, as indicated in the figure legends; p < 0.05 was considered significant after adjusting for multiple testing where indicated in the figure legends.

## Results

### Loss of Acer1 leads to increased ceramide levels in the skin, abnormal hair and cyclic alopecia

**Figure 1:**
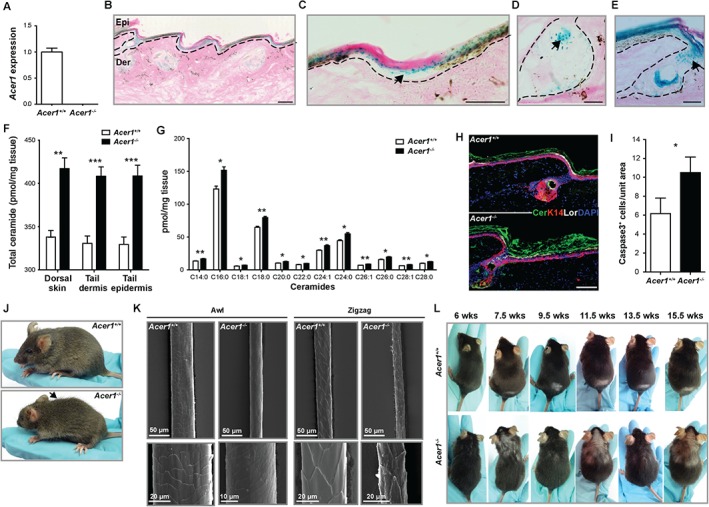
Mice lacking Acer1 show altered ceramide levels in the skin, hair shaft abnormalities and cyclic alopecia. (A) RT–qPCR analysis of Acer1 expression in tail skin of wild‐type and homozygous mice (n = 4 Acer1 ^+/+^, n = 3 Acer1 ^−/−^ females at age 7 weeks). (B–E) LacZ reporter staining shows specific expression of Acer1 in (B) differentiated epidermal layers (Acer1^+/−^), (C) interfollicular epidermis (arrow; Acer1^+/−^), (D) sebaceous glands (arrow; Acer1^+/−^) and (E) infundibulum (arrow; Acer1^−/−^); Epi, epidermis; Der, dermis. (F) Total ceramide content of dorsal skin, tail epidermis and tail dermis and (G) determination of the individual ceramide species in dorsal skin; data are mean ± standard error (SE) of the mean (n = 5 males, aged 9 weeks) per tissue per genotype; statistical analysis is unpaired t‐test, with adjustment for multiple testing for the individual ceramide species using the Holm–Sidak method with α set to 5%; *p < 0.05, **p < 0.01, ***p < 0.001, ****p < 0.0001. (H) Expression of ceramide (Cer), epidermal basal layer marker keratin 14 (K14) and differentiated layer marker loricrin (Lor) in wild‐type (WT) and Acer1^−/−^ tail epidermis, with nuclear staining (blue) by DAPI (n = 2 males at age 28 weeks/genotype with three technical replicates). (I) Analysis of immunostained skin sections from 28 week‐old Acer1^−/−^ mice shows increased numbers of caspase3^+^ cells compared to wild‐type mice; data are mean ± SE (n = 2 males/genotype with three technical replicates), statistical analysis was unpaired t‐test, *p = 0.0346. (J) Acer1 ^−/−^ mice showed obvious dorsal coat abnormalities (mixed length or long hair, arrow) at age 6 weeks. (K) Representative scanning electron microscopy (SEM) images of different hair types (Awl and Zigzag) from wild‐type and Acer1^−/−^ mice at age 30 weeks (n = 2/genotype). (L) Hair loss associated with hair cycle and age: the small denuded area seen on the lower dorsum of the Acer1 ^+/+^ mouse from 7.5 weeks is the region shaved to perform the hair follicle cycling analysis; this patch has completely regrown in the Acer1 ^−/−^ mouse by 7.5 weeks. Scale bars = 100 µm (B–E, H)

To understand the function of Acer1 in the skin, we used Acer1^tm1a(EUCOMM)Wtsi^ knockout mice (hereafter referred to as Acer1 ^−/−^ mice) generated by the Sanger Institute Mouse Pipelines 7, 11. Reverse transcription–quantitative PCR (RT–qPCR) confirmed a loss of Acer1 expression in the homozygotes (Figure 1A). The skin of these mice showed an increased level of mRNA for the acid ceramidase (Asah1) but not for any of the other alkaline ceramidases or the ceramide synthases (see supplementary material, Figure S1). LacZ reporter staining showed strong expression of Acer1 in the granular layer of interfollicular epidermis (Figure 1B), in agreement with previous studies that demonstrated the essential role of ACER1 in keratinocyte differentiation 12. However, strong expression was also observed in the sebaceous glands and infundibulum (Figure 1B–E). As expected, Acer1^−/−^ mice showed a significant increase in total ceramide levels in the dorsal skin, tail epidermis and dermis relative to wild‐type controls (Figure 1F). The increased ceramide levels were observed in all ceramide species (Figure 1G; see also supplementary material, Table S1), with the greatest increase in C16:0, and not restricted to long‐chain ceramides, as previously reported to be the preferred substrate for ACER1 5, 12. The increased ceramide levels were also visualized by immunostaining with an anti‐ceramide antibody, which showed strong staining within the stratum corneum (Figure 1H), and there was a significantly increased number of apoptotic (cleaved caspases‐3‐positive) cells in the skin of Acer1^−/−^ mice relative to wild‐type (Figure 1I). Quantification of ceramide content in the stratum corneum of Acer1^−/−^ mice showed an increased total mean fluorescence intensity relative to wild‐type mice (see supplementary material, Figure S2A). As expected, the increase in ceramide was accompanied by decreases in sphingosine, sphingosine‐1‐phosphate, dihydrosphingosine and dihydrosphingosine‐1‐phosphate levels (see supplementary material, Figure S2B). Concomitant with this were significant increases in levels of monohexosylceramides and sphingomyelins (see supplementary material, Figure S2C), dihydroceramides, monohexodihydrosylceramides and dihydrosphingomyelin (Figure S2D), phytoceramides and monohexosylphytoceramides (Figure S2E) and hydroxyacylceramides, monohexosylhydroxyacylceramides and hydroxyacylsphingomyelin (Figure S2F). Previous in vitro studies have reported that ACER1 fails to hydrolyse any dihydroceramides or phytoceramides 5, thus further investigations would be needed to determine whether the increase in levels of these lipids observed in the skin of Acer1^−/−^ mice is a direct result of lack of ACER1‐mediated hydrolysis or some indirect/compensatory effect.

*Acer1* ^−/−^ mice were viable and fertile, with a normal lifespan. However, dorsal coat abnormalities (consisting of mixed length or long hair) were observed at P25 (see supplementary material, Figure S3) and became more obvious by age 6 weeks (Figure 1J). Dysmorphology analysis performed at age 10 weeks showed abnormalities in the hair coverage in *Acer1^−/−^* mice, with the abnormalities being more severe in males (see supplementary material, Table S2). SEM of different hair types showed that, unlike the scaly tile arrangement of the cuticle that covers the surface of wild‐type hairs, *Acer1^−/−^* hair shafts showed compressed and smooth cuticles in awl hairs, and a rough and irregular surface in *Acer1^−/−^* zigzag hair shaft cuticles (Figure 1K).

Whole‐mount analysis of tail epidermis showed alterations in the hair follicle (HF) patterning of *Acer1^−/−^* epidermis, with disruption in the arrangements of the HF triplet clusters (see supplementary material, Figure S4). There was no evidence of altered hair follicle cycling at age 6 weeks, as similar numbers of wild‐type (22/23) and *Acer1^−/−^* (21/22) mice were in the anagen phase. However, follow‐up 4 weeks later showed that all *Acer1* ^−/−^ mice had regrown the hair following shaving, compared to only one of the wild‐type mice, suggesting that the pattern of hair growth in *Acer1* ^−/−^ mice is disrupted. Indeed, frequent imaging of *Acer1* ^−/−^ mice at age 6–16 weeks demonstrated a pattern of cyclical, sporadic hair loss followed by regrowth (Figure 1L), consistent with cyclic alopecia.

### Acer1^−/−^ mice show hyperproliferation, inflammation and abnormal differentiation of the epidermis

**Figure 2:**
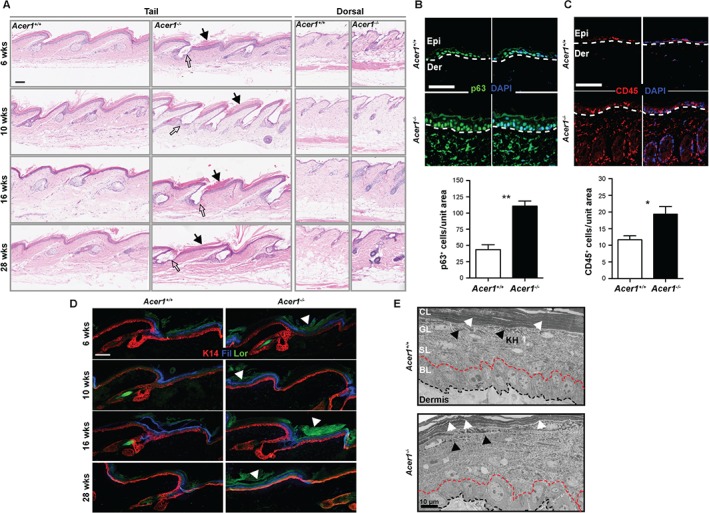
The skin of Acer1^−/−^ mice shows hyperproliferation, inflammation and abnormal differentiation. (A) Representative image of H&E staining of tail at different ages (n = 3/age and genotype); open arrows, abnormal SGs in Acer1 ^−/−^ skin. (B) Note thickened differentiated layers of the epidermis indicated by black arrows. Immunostaining of skin sections from 27 week‐old Acer1^−/−^ mice shows increased numbers of p63^+^ cells compared to wild‐type mice; data are shown as mean ± SE (n = 3) and analysed using unpaired t‐test (**p = 0.0017); a representative image is shown; green, p63 staining; blue, nuclear staining by DAPI; Epi, epidermis; Der, dermis. (C) Immunostaining of skin sections from 27 week‐old Acer1^−/−^ mice shows increased numbers of CD45^+^ cells compared to wild‐type mice; data are shown as mean (n = 3) ± SE and analysed using unpaired t‐test (**p = 0.0216); a representative image is shown; red, CD45 staining; blue, nuclear staining by DAPI; Epi, epidermis; Der, dermis. (D) Expression of epidermal basal layer marker Keratin 14 (K14) and differentiated layer markers filaggrin (Fil) and loricrin (Lor) in Acer1^−/−^ epidermis from different age points (n = 3/age and genotype). (E) TEM images of the epidermal layers: white arrows, abnormal arrangement of cornified layers; black arrows, abnormal keratohyalin (KH) granules in granular layer of Acer1^−/−^ epidermis when compared to wild‐type epidermis; BL, basal layer; SL, suprabasal layer; GL, granular layer; CL, cornified layer; scale bars = 100 µm except in (E) 10 µm

Dysmorphology analysis of the skin performed at age 10 weeks revealed a significantly increased incidence of dry/scaly skin in the *Acer1^−/−^* mice relative to wild‐type (see supplementary material, Table S2). Histological examination of H&E‐stained dorsal and tail skin at age 6, 10, 16 and 27–29 weeks revealed that, compared with wild‐type mice, *Acer1^−/−^* mice showed hyperproliferation of both the dorsal and tail epidermis. Increased areas of terminally differentiated (cornified) cells and thickened dermis were also observed, as measured by a mildly increased dermal thickness at 16 weeks, seen mostly in the tail skin and less so in the dorsal skin (Figure 2A; see also supplementary material, Figure S5). Consistent with this, an increased number of cells expressing p63, a marker for basal layer keratinocytes with proliferative capacity, were found in the dermis and epidermis of *Acer1^−/−^* mice when compared to wild‐type (Figure 2B). Increased levels of inflammatory infiltrate were also present in the skin of *Acer1^−/−^* mice relative to controls, as shown by the number of cells positive for the pan‐leukocyte marker CD45 (Figure 2C).

To determine whether there were any defects in epidermal differentiation in *Acer1^−/−^* mice, we performed immunostaining for K14 (basal layer), filaggrin (granular layer) and loricrin (cornified layer) in tail skin of *Acer1^−/−^* and wild‐type mice at 6, 10, 16 and 28 weeks. There were no obvious changes in the basal and granular layers of the epidermis, although *Acer1^−/−^* mice showed a strong increase in the thickness of the cornified layer relative to controls (Figure 2D). We further analysed *Acer1^−/−^* skin by TEM to determine whether there were any obvious ultrastructural defects in the integrity of the epidermal layers. Electron micrographs showed similar arrangements of the cornified layer, although *Acer1^−/−^* mice had an abnormal organization of the granular‐transitional cell layer junctional area and differences in the sizes of the keratohyalin (KH) granules in the granular layer relative to wild‐type mice (Figure 2E).

### Loss of Acer1 results in sebaceous gland abnormalities and infundibulum expansion

**Figure 3:**
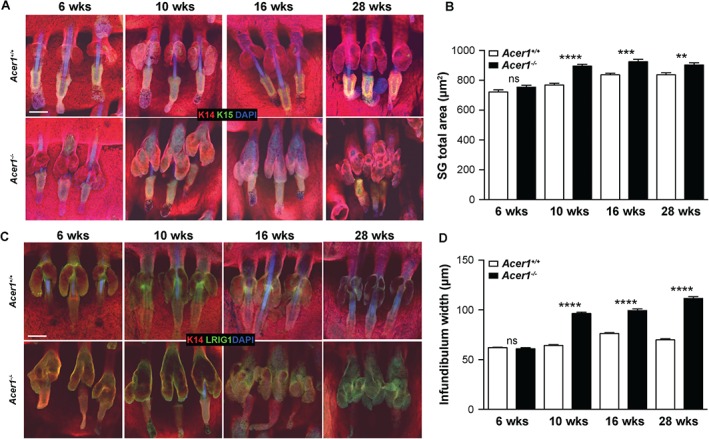
Sebaceous gland and infundibulum expansion in Acer1^−/−^ mice. (A) Representative epidermal whole mount immunostaining of K14 (red) and K15 (green) markers at different ages; blue, DAPI; n = 3/age and genotype). (B) Quantification of total SG area. (C) Representative epidermal whole mount immunostaining of the sebaceous–hair follicle junctional zone marker Lrig1 (green); red, K14; blue, DAPI; n = 3/age and genotype). (D) Quantification of infundibulum width; data are shown as mean ± SE (n = 3) and analysed using unpaired t‐test; ns, not significant; **p = 0.0013, ***p = 0.0010, ****p < 0.0001; scale bars = 100 µm

Histological examination of H&E‐stained dorsal and tail skin at age 6, 10, 16 and 28 weeks showed that *Acer1^−/−^* mice had abnormal sebaceous glands (SGs) relative to wild‐type mice (Figure 2A). Immunostaining of epidermal whole‐mounts with antibodies to K14 and K15 13 at these time points showed progressive structural changes in *Acer1^−/−^* SGs (Figure 3A), a site of high‐level *Acer1* expression (Figure 1D, E). There was a gradual increase in the size of SGs and the width of the infundibulum from age 10 weeks, with SGs starting to fuse with the infundibulum by 16 weeks (Figure 3A, B). There was multiplication and enlargement of SG lobules by 28 weeks. Immunostaining for Lrig1, a marker of HF‐SG junctional zone stem cells*,* showed that Lrig1 expression expanded beyond the junctional zone in *Acer1^−/−^* HFs (Figure 3C) 14. We also observed abnormalities in the HF bulge in 28 week‐old *Acer1^−/−^* mice after immunostaining for K15, a marker of the HF bulge, where slow‐cycling stem cells reside (Figure 3A) 15.

**Figure 4:**
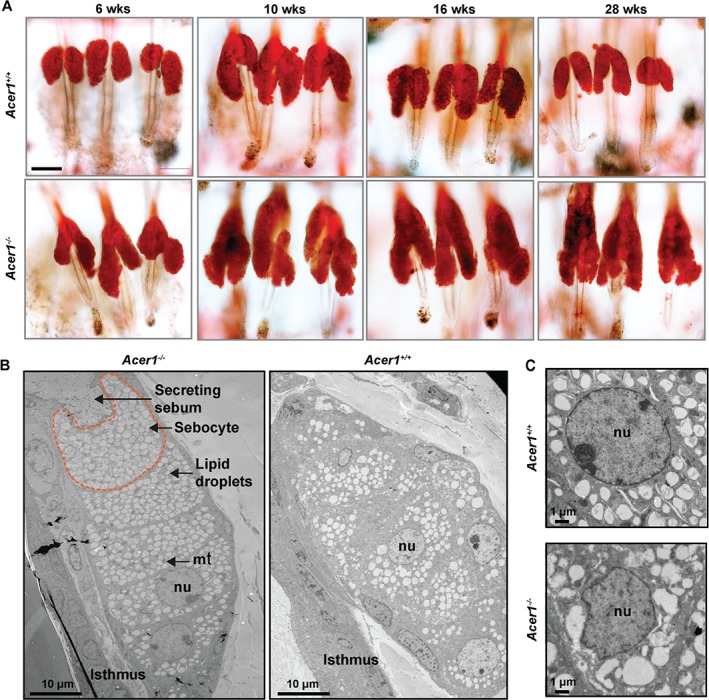
Sebaceous gland abnormalities in Acer1^−/−^ mice. (A) Representative oil red O staining of epidermal whole mounts at different ages (n = 3/age and genotype). (B, C) TEM images of the SG ultrastructure: note the irregular lipid droplets and nuclear structure in Acer1^−/−^ sebocytes; scale bars = 100 µm, unless stated otherwise

We observed oil red O staining neutral lipids extending into the *Acer1^−/−^* infundibulum with age (Figure 4A), confirming the striking abnormalities seen in the SGs of *Acer1^−/−^* mice. Immunohistochemical examination of the expression of enzymes critical for lipid synthesis in maturing sebocytes, such as fatty acid synthase (FASN) and peroxisome proliferator activated receptor‐γ (PPARγ), showed unexpected localization of PPARγ in the cornified layer of the *Acer1^−/−^* epidermis, whereas FASN expression was unchanged between *Acer1^−/−^* and wild‐type (see supplementary material, Figure S6). Further, ultrastructural, analysis of SGs revealed a striking difference in sebocyte arrangement, lipid droplet structures and nuclear shapes in *Acer1^−/−^* SGs compared to wild‐type (Figure 4B, C).

### Acer1 ^−/−^ mice display increased transepidermal water loss and hypermetabolism with associated reduction of fat content during aging

**Figure 5:**
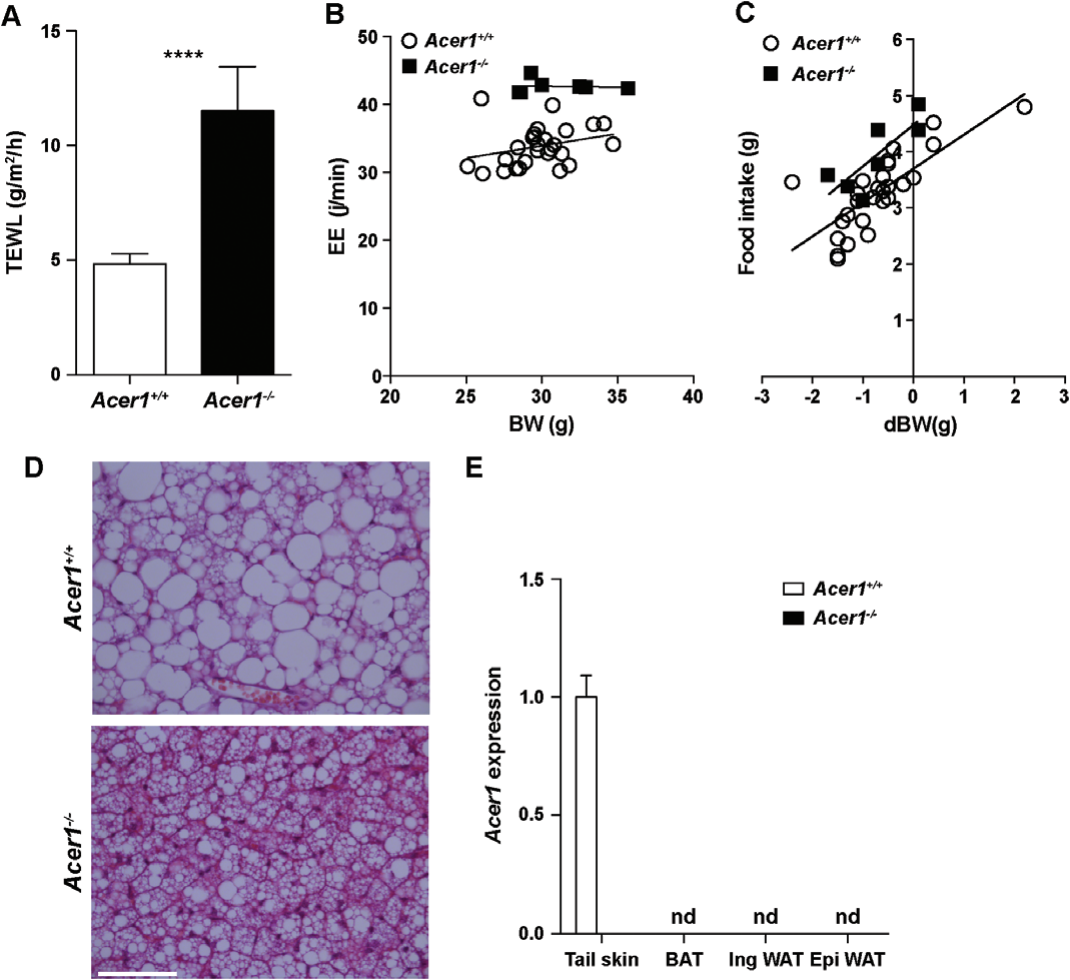
Increased transepidermal water loss and hypermetabolism of Acer1^−/−^ mice. (A) Transepidermal water loss (TEWL) in 19 week‐old male mice (n = 5 Acer1^+/+^, n = 5 Acer1^−/−^); data are shown as mean ± SE and analysed using unpaired t‐test; ****p < 0.0001. (B) Energy expenditure (EE; estimated EE for a 30 g mouse, Acer1 ^−/−^ versus Acer1 ^+/+^ = 42.7 j/min versus 33.8 j/min; ANCOVA corrected for body weight, p = 5.7 × 10^−9^) and (C) food intake [food intake at zero body weight change (dBW): Acer1 ^−/−^ versus Acer1 ^+/+^ = 4.5 ± 0.2 g versus 3.7 ± 0.1 g; ANCOVA corrected for change in body weight, p = 0.00116] were measured using indirect calorimetry for 22 h (n = 27 Acer1 ^+/+^, n = 7 Acer1 ^−/−^ males). (D) Representative image of H&E‐stained sections of BAT from 18 week‐old male mice (n = 3/genotype); scale bar = 50 µm. (E) Gene expression of Acer1 in adipose tissue depots from 18 week‐old male mice (n = 3/genotype): tail skin RNA from Figure 1A was reused as a positive control for Acer1 detection; nd, not detected. Mean control gene (B2m) C t values for each tissue were: tail skin, 26.9; inguinal WAT, 19.8; epididymal WAT, 19.5; BAT, 22.1

To determine whether there were any defects in the integrity of the skin, we performed a barrier function examination based on dye exclusion assays at E16.5–18.5. Whilst initiation of epidermal barrier formation was slightly delayed in *Acer1^−/−^* embryos, the barrier was fully formed and indistinguishable from wild‐type by late E17.5–18.5 (ie no dye penetration into the skin at this time point; see supplementary material, Figure S7). As the skin serves as a barrier to water loss, we next examined the evaporation rate from skin and found that *Acer1^−/−^* mice showed significantly increased levels of transepidermal water loss (TEWL) relative to controls (Figure 5A). UDP‐glucose:ceramide glucosyltransferase (*Ugcg*)‐deficient and elongation of very long chain fatty acids (*Elovl1*)‐deficient mice, which show elevated levels of various ceramide species in the skin, also show increased levels of TEWL 16, 17. As the skin is also a major site of thermoregulation, we examined *Acer1* mice for evidence of abnormal metabolic control. Indirect calorimetry analysis showed that *Acer1* ^−/−^ mice had a 26% increase in energy expenditure (EE) compared to wild‐type mice (Figure 5B). Matching this, there was a 21% increase in food intake in *Acer1* ^−/−^ mice during the period of study (Figure 5C), although similar increases in oxygen consumption and carbon dioxide production resulted in an unchanged respiratory exchange ratio (see supplementary material, Figure S8A). Total activity also increased by 24% in *Acer1^−/−^* mice (Figure S8B).

We hypothesized that elevated EE in *Acer1* ^−/−^ mice may result in long‐term consequences for body weight and composition. At 9 weeks of age body weights and adipose tissue depot weights were normal, except for a small increase in epididymal white adipose tissue (WAT) in *Acer1* ^−/−^ mice (see supplementary material, Figure S8C–F). In contrast, older (31–34 week‐old) *Acer1* ^−/−^ mice had a lower body weight and significantly less WAT and brown adipose tissue (BAT) relative to controls (Figure S8C–F), which is consistent with a long‐term alteration in energy balance. Histological examination of BAT from *Acer1* ^−/−^ mice showed smaller lipid vacuoles within the brown adipocytes and mildly increased eosinophilia compared with the controls (Figure 5D). These alterations in BAT were not associated with altered levels of Ucp1, the mitochondrial transporter protein required for heat generation by non‐shivering thermogenesis (see supplementary material, Figure S7G, H), and Ucp1 protein was not detectable in inguinal WAT from *Acer1^−/−^* mice (data not shown). Moreover, *Acer1* expression is absent even in *Acer1* ^+/+^ WAT and BAT, reinforcing that the hypermetabolic phenotype is unlikely to be a primary defect of adipose tissues (Figure 5E). Considering the skin phenotype seen in these mice, the combination of elevated EE and food intake is likely indicative of enhanced metabolic activity for heat production due to reduced thermal insulation, rather than a primary metabolic disorder.

## Discussion

Ceramides are the major sphingolipid components of lamellar sheets present in the intercellular spaces of the stratum corneum and provide the barrier property of the epidermis 18. ACER1 is known to be a key enzyme in ceramide metabolism 19, 20. In this study, we showed that Acer1 is expressed in the differentiating layer of the epidermis and SGs and that targeted deletion of *Acer1* leads to increased levels of ceramides in the skin and striking postnatal skin phenotypes (Figure 1B–I). Abnormalities in *Acer1^−/−^* skin include progressive hair loss and abnormal hair shafts (Figure 1J); disruption in hair shaft cuticle differentiation is known to cause cyclic alopecia 21. The alopecia‐like phenotype and the obvious hair shaft cuticular abnormalities (Figure 1I) indicate that *Acer1* is indispensable for the formation of the hair shaft cuticle and thus for maintaining hair function. It has also been reported that modulation of human *ACER1* mediates differentiation in epidermal keratinocytes 5. As shown in this study, *Acer1*‐deficient mice show abnormalities in the terminally differentiated cornified layer, indicating a key role for this enzyme in normal epidermal differentiation (Figure 2D).

Along with the epidermis, the SGs play an important role in maintaining the integrity of the skin through the production of lipid‐rich sebum by fully differentiated sebocytes 22. Our observation of the specific expression of *Acer1* in SGs and the striking SG abnormalities following *Acer1* deletion that manifest in the expansion of glandular and infundibular compartments (Figures 3, 4A), as well as the altered sebocyte and lipid droplet structures (Figure 4B, C), delineates a role for Acer1 in maintaining SG integrity. As the skin is a heterogeneous organ, investigation of the ceramide composition within specific skin organelles and compartments, such as the sebaceous glands and stratum corneum, would provide further insight into the key role of lipids in maintaining skin homeostasis.

Keratin 15‐positive cells within the HF bulge stem cell population and *Lrig1*‐positive multipotent stem cells in the junctional zone are essential for maintaining epidermal homeostasis 14, 15. Our observations that these two stem cell compartments expand in *Acer1^−/−^* epidermis (Figure 3D) indicate a possible role of Acer1 in regulating cell fate within the epidermis. Interestingly, recent studies have shown that ceramide synthase 4 (CerS4)‐deficient mice also show an expanded stem cell (Lrig1‐positive) population, with an expanded HF domain and SG enlargement 23, 24. Whilst the skin of the CerS4‐deficient mice contains less C20‐containing sphingolipid, several ceramide species are increased in abundance 24. This would suggest that epidermal stem cells are exquisitely sensitive to the concentration of specific ceramide species in the skin. Collectively, our data support the involvement of ACER1 in maintaining tissue homeostasis by regulating the differentiation programme of multiple compartments within murine epidermis.

The skin and hair have well‐established roles in regulating water loss, thermal homeostasis and maintaining core body temperature. A number of mouse models have been described that display the combination of a hair/skin phenotype together with stimulation of EE and/or BAT activity 25. Similarly, we found that *Acer1^−/−^* mice display a striking increase in TEWL and EE, and also that older *Acer1^−/−^* mice have both lower body weights and smaller adipose tissue depots compared to wild‐type mice, indicative of a disturbance in energy balance, despite the fact that *Acer1* is not expressed in either WAT and BAT tissues. Combining the hair and skin phenotype with the predominant expression of *Acer1^−/−^* in the epidermis, *Acer1^−/−^* mice are likely to have a primary deficiency in thermal insulation that in turn requires enhanced energy expenditure and heat production to maintain body temperature. Interestingly, we found no evidence that *Acer1^−/−^* BAT has increased levels of UCP1, or the so‐called 'browning' of WAT in *Acer1^−/−^* mice, since UCP1 remains undetectable in inguinal WAT. This suggests that other mechanisms, such as direct functional activation of UCP1, may be occurring to promote energy expenditure downstream of the primary defect in skin function.

In summary, our study defined the *in vivo* role of Acer1 in the epidermis, hair shaft cuticle and SGs of mice. The phenotype of *Acer1^−/−^* mice suggests that the primary involvement of Acer1 is in maintaining tissue homeostasis by facilitating the differentiation programme of various compartments within the murine epidermis, and its secondary role is in regulating transepidermal water loss and whole‐body energy homeostasis.

## Author contributions

KL‐A, VEV, CJL, DL, HJP, AG, AG, DG, ER, LG and GV‐B generated data; KL‐A, CJL, AOS, CI, AG, AG, NAK, MJA, TB, SS, DJA, FMW and LvDW analysed and interpreted the data; SS oversaw the mass spectrometry analysis; DJA, FMW and LvdW supervised the project; KL‐A, VEV, CJL, AOS, CI, AG, AG and LvdW generated manuscript figures; and KL‐A, CJL, AOS and LvdW wrote the manuscript, with the input and approval of all authors.

**SUPPLEMENTARY MATERIAL ON THE INTERNET**

The following supplementary material may be found in the online version of this article:

Supplementary materials and methods

Figure S1. RT–qPCR analysis of the levels of the ceramidases and ceramide synthases expressed in the skin of wild‐type and *Acer1^−/−^* mice relative to the endogenous control *B2m*

Figure S2. Altered lipid composition of skin from *Acer1^−/−^* mice

Figure S3. *Acer1* ^−/−^ pups have normal onset of hair growth

Figure S4. Altered hair follicle patterning in *Acer1^−/−^* epidermis

Figure S5. *Acer1^−/−^* mice have an altered skin phenotype

Figure S6. Expression of sebaceous gland differentiation markers in *Acer1* epidermis

Figure S7. Normal epidermal barrier in *Acer1^−/−^* embryos

Figure S8. Characterization of the hypermetabolic phenotype in *Acer1* ^−/−^ mice

Table S1. Altered lipid composition of skin from *Acer1^−/−^* mice

Table S2. Dysmorphology analysis of 10 week‐old *Acer1* ^−/−^ and wild‐type mice

Table S3. Dysmorphology parameters

## Supporting information

Appendix S1. Supplementary material

RT–qPCR analysis of the levels of the ceramidases and ceramide synthases expressed in the skin of wild‐type and Acer1^–/–^ mice relative to the endogenous control B2m (n = 4 Acer1 ^+/+^, n = 3 Acer1 ^–/–^ females at age 7 weeks); Asah2 and CerS1 are not shown, as they are not expressed in mouse skin. Data are shown as mean ± SE/genotype; statistical analysis was by unpaired t‐test with adjustment for multiple testing for the individual enzymes species, using the Holm–Sidak method with α set to 5%; *** p = 0.0004

Altered lipid composition of skin from Acer1^–/–^ mice. (A) Quantification of ceramide content in the stratum corneum (SC) from 28 week‐old Acer1^–/–^ mice shows increased total mean fluorescence intensity; data are mean ± SE (n = 2 males/genotype with two technical replicates); statistical analysis was by unpaired t‐test; *p = 0.0263. (B) Sphingosine, sphingosine‐1‐phosphate, dihydrosphingosine and dihydrosphingosine‐1‐phosphate. (C) Monohexosylceramides and sphingomyelins. (D) Dihydroceramides, monohexosyldihydroceramides and dihydrosphingomyelin. (E) Phytoceramides, hexosylphytoceramides, phytosphingomyelin and phytosphingosine. (F) Hydroxyacylceramides (which includes both 2‐hydroxy and omega‐O‐acylceramide), monohexosylhydroxyacylceramides and hydroxyacylsphingomyelin; data are mean ± SE (five males, aged 9 weeks)/tissue/genotype; statistical analysis was by unpaired t‐test with adjustment for multiple testing for the individual ceramide species, using the Holm–Sidak method with α set to 5%; *p < 0.05, **p < 0.01

Acer1 ^–/–^ pups have normal onset of hair growth. Dorsal images of Acer1 ^+/+^ and Acer1 ^–/–^ male pups taken 5–25 days postpartum, demonstrating the similarity in the timing of hair growth between genotypes. By P25, Acer1 ^–/–^ male display the abnormal hair length phenotype

Altered hair follicle patterning in Acer1^–/–^ epidermis. Representative images of tail epidermal whole mount with K14 and K15 staining, showing irregular arrangement of hair follicle triplet clusters in Acer1^–/–^ epidermis when compared to wild‐type (n = 3/age and genotype); scale bar = 100 µm

Acer1^–/–^ mice have an altered skin phenotype. Quantification of dorsal dermis thickness at different ages; data are shown as mean ± SD (n = 3) and analysed using unpaired t‐test; ns, not significant; **p = 0.0013, ***p = 0.0010, ****p < 0.0001

Expression of sebaceous gland differentiation markers in Acer1 epidermis. Representative immunostaining of skin sections from 16 week‐old mice with anti‐FASN shows no difference in expression between wild‐type and Acer1^–/–^ mice, whereas ectopic expression of PPARγ is evident in Acer1^–/–^ compared to wild‐type epidermis (n = 3/age and genotype); scale bar = 100 µm

Normal epidermal barrier in Acer1^–/–^ embryos. Barrier‐dependent toluidine blue dye exclusion assay on E16.5–18.5 Acer1 embryos shows that the dye fails to penetrate E17.5 embryos, indicating the formation of a fully functional skin barrier

Characterization of the hypermetabolic phenotype in Acer1 ^–/–^ mice. (A) Respiratory exchange ratio (RER) and (B) total spontaneous activity were measured using indirect calorimetry for 22 h (n = 27 Acer1 ^+/+^, n = 7 Acer1 ^–/–^ males). (C–F) Mice aged 9 and 32 weeks were weighed and culled to dissect out adipose tissue depots (n = 5/time point and genotype). (G) Quantitation of total UCP1 content/intrascapular BAT depot and (H) western blot images of BATs from 9 and 32 week‐old wild‐type and Acer1 ^–/–^ mice (n = 5/time point and genotype). Vinculin was used as the loading control; data are shown as mean ± SD and were analysed within each time point using Mann–Whitney test; ns, not significant; **p < 0.01

Altered lipid composition of skin from Acer1‐/‐ mice.

Dysmorphology analysis of 10 week‐old Acer1 ^–/–^ and wild‐type mice

Dysmorphology parameters
