# Supplementary material for: Alkaline ceramidase 1 is essential for mammalian skin homeostasis and regulating whole‐body energy expenditure
Source: J Pathol. 2016 May 30;239(3):374–83. doi: 10.1002/path.4737 (PMC4924601; doi:10.1002/path.4737)
Supplement: Supplementary file 1 — Appendix S1. Supplementary material [file PATH-239-374-s011.doc]

**<Supplementary material>**

+A: **Supplementary materials and methods**

+B: Mice

For qPCR analysis, RNA from tail skin was extracted from 7 week-old female mice and subjected to RT–qPCR using an RNA-to-Ct One-Step Kit (Life Technologies, Carlsbad, CA, USA), using TaqMan assay for mouse *Acer1* (Mm00460332_m1), *Acer2* (Mm00519876_m1), *Acer3* (Mm00502940_m1), *Asah1* (Mm00480021_m1), *CerS2* (Mm01258345_g1), *CerS3* (Mm03990709_m1), *CerS4* (Mm00482658_m1), *CerS5* (Mm00510998_m1), *CerS6* (Mm00556165_m1) and a *B2m* primer-limited endogenous control (Mm00437762_m1; all from Life Technologies) to normalize for variations between the amounts of input RNA. Reactions were performed in triplicate using a ViiA 7 qPCR machine (Life Technologies) and analysed using the 2^–^*^ΔΔC^*^t^ method [26]. Housing and husbandry conditions were as described previously [27] with the exceptions that mice were maintained on Mouse Breeders Diet (Lab Diets, 5021–3) throughout the study and, when necessary (due to welfare concerns), the mice were individually housed. Cages were processed randomly and different genotypes could be housed together; however, blinding was not possible, due to the obvious phenotypes of the homozygotes.

+B: Histology, whole-mount staining and imaging

Primary antibodies were: directly conjugated (AlexaFluor 555) K14 (LL002, in house) or rabbit anti-K14 (Covance; PRB-155P, 1:100 dilution); directly conjugated (AlexaFluor 488) K15 (LHK-15; in-house, 1:50 dilution); rabbit anti-filaggrin (Covance; PRB-417P, 1:100 dilution); rabbit anti-loricrin (Covance; PRB-145P, 1:100 dilution); rabbit anti-PPARγ (Cell Signaling; 2435s, 1:200 dilution); mouse anti-FASN (SCBT; sc48357. 1:100 dilution); mouse anti-CD45 (BD Pharmingen; clone 30-F11, 1:100 dilution); human anti-p63 (SCBT; sc367333, 1:100 dilution); mouse anti-ceramide (Glycobiotech GmbH; clone S58-9, 1:100 dilution) and rabbit anti-active-caspase 3 (R&D Systems; AF835, 1:500 dilution). AlexaFluor (Life Technologies) dye-conjugated secondary antibodies were used at 1:250 dilution. For oil red O staining, tail epidermal sheets were fixed in 4% PFA for 1 h at room temperature and washed with PBS. The sheets were then incubated for 10 min at room temperature with 60% filtered oil red O stock solution (0.3 g/100 ml isopropanol) in a 24-well plate. The epidermal sheets were washed with 60% v/v isopropanol and then water before mounting on slides. Visualization was performed using a light microscope. Epidermal whole-mount staining was performed as described previously [13]. Nikon A1 or A1R upright confocal microscopes were used for image acquisition and quantification was performed using either Fiji (ImageJ) or Icy v. 1.7.3.0 software.

+B: Embryo whole-mount dye-penetration assay

Embryos were quickly dehydrated and rehydrated through a methanol series for 1 min on ice and rinsed in PBS. Staining was performed by submersion in 1% toluidine blue in water. The embryos were destained in PBS, pH 7.4, until the pattern appeared. The embryos were imaged using a Leica M205C microscope and LAS4.4 Leica software.

+B: Electron microscopy

For scanning electron microscopy (SEM) analysis, hair samples were mounted on double-sided adhesive carbon tape over aluminium pin stubs before gold coating. Samples were examined and images recorded using an FEI Quanta 200F field emission scanning electron microscope operated at 10 kV in high-vacuum mode. For transmission electron microscopy (TEM) analysis, skin samples were fixed overnight at 4°C with 2% w/v paraformaldehyde, 2.5% v/v glutaraldehyde in 0.15 m cacodylate buffer, pH 7.4, and postfixed in 1.3% w/v osmium tetroxide in 0.15 m cacodylate buffer, pH 7.4, for 1 h. Samples were then stained *en bloc* with 2% uranyl acetate in 50% ethanol for 1 h at room temperature and dehydrated through a graded ethanol series, before infiltration with TAAB epoxy resin and polymerization at 70°C for 24 h. Ultrathin sections (50–70 nm) were prepared using a Reichert–Jung Ultracut E ultramicrotome, mounted on slot grids and contrasted using uranyl acetate and lead citrate. Samples were examined on a FEI Tecnai 12 transmission microscope operated at 120 kV. Images were acquired with an AMT 16000M camera.

+B: Adipose tissue analysis

Intrascapular brown adipose tissues (iBATs) were isolated from either young (9 week-old) or old (31–34 week-old) *Acer1*^+/+^ and *Acer1*^–/–^ male mice (*n =* 5/genotype) and weighed. Protein extracts were generated from weighed portions of intrascapular BATs by homogenization in ice-cold T-PER protein extraction reagent (Pierce, Rockford, IL, USA) containing a protease/phosphatase inhibitor cocktail (Halt, Thermo Scientific, USA), using a single-use pestle in a 1.5 ml Eppendorf tube (VWR, Radnor, PA, USA). Homogenates were centrifuged (4°C, 20 000 × *g* for 15 min) and the protein content of the lysate quantitated using BCA Assay Reagent (Pierce). Protein aliquots (45 μg) were assayed by western blot, separating the samples on 4–12% Bis–Tris gels (Life Technologies, Paisley, UK). Primary antibodies for were Ucp1 (Abcam; ab10983, 1:1000 dilution) and loading control vinculin (Abcam; ab129002, 1:10000 dilution). A goat anti-rabbit IgG HRP-conjugate was used as a secondary antibody (Millipore, Billerica, MA, USA). The blots were imaged by enhanced chemiluminescence and bands quantitated using an ImageLAS 4000 system (GE Healthcare, Chalfont St Giles, UK). These values were converted to total iBAT UCP1 content by accounting for total extractable protein (using the BCA assay value) and the total iBAT weight, with the final data presented relative to *Acer1*^+/+^ levels for each time point.

**<Supplementary figures>**

**Figure S1.** RT–qPCR analysis of the levels of the ceramidases and ceramide synthases expressed in the skin of wild-type and *Acer1^–/–^* mice relative to the endogenous control *B2m* (*n =* 4 *Acer1*^+/+^, *n =* 3 *Acer1*^–/–^ females at age 7 weeks); *Asah2* and *CerS1* are not shown, as they are not expressed in mouse skin. Data are shown as mean ± SE/genotype; statistical analysis was by unpaired *t*-test with adjustment for multiple testing for the individual enzymes species, using the Holm–Sidak method with α set to 5%; *** *p =* 0.0004

**Figure S2.** Altered lipid composition of skin from *Acer1^–/–^* mice. (A) Quantification of ceramide content in the stratum corneum (SC) from 28 week-old Acer1^–/–^ mice shows increased total mean fluorescence intensity; data are mean ± SE (*n =* 2 males/genotype with two technical replicates); statistical analysis was by unpaired *t*-test; **p =* 0.0263. (B) Sphingosine, sphingosine-1-phosphate, dihydrosphingosine and dihydrosphingosine-1-phosphate. (C) Monohexosylceramides and sphingomyelins. (D) Dihydroceramides, monohexosyldihydroceramides and dihydrosphingomyelin. (E) Phytoceramides, hexosylphytoceramides, phytosphingomyelin and phytosphingosine. (F) Hydroxyacylceramides (which includes both 2-hydroxy and omega-*O*-acylceramide), monohexosylhydroxyacylceramides and hydroxyacylsphingomyelin; data are mean ± SE (five males, aged 9 weeks)/tissue/genotype; statistical analysis was by unpaired *t*-test with adjustment for multiple testing for the individual ceramide species, using the Holm–Sidak method with α set to 5%; **p <* 0.05, ***p <* 0.01

**Figure S3.** *Acer1*^–/–^ pups have normal onset of hair growth. Dorsal images of *Acer1*^+/+^ and *Acer1*^–/–^ male pups taken 5–25 days postpartum, demonstrating the similarity in the timing of hair growth between genotypes. By P25, *Acer1*^–/–^ male display the abnormal hair length phenotype

**Figure S4.** Altered hair follicle patterning in *Acer1^–/–^* epidermis. Representative images of tail epidermal whole mount with K14 and K15 staining, showing irregular arrangement of hair follicle triplet clusters in *Acer1^–/–^* epidermis when compared to wild-type (*n =* 3/age and genotype); scale bar = 100 μm

**Figure S5.** *Acer1^–/–^* mice have an altered skin phenotype. Quantification of dorsal dermis thickness at different ages; data are shown as mean ± SD (*n =* 3) and analysed using unpaired *t-*test; ns, not significant; ***p =* 0.0013, ****p =* 0.0010, *****p <* 0.0001

**Figure S6.** Expression of sebaceous gland differentiation markers in *Acer1* epidermis. Representative immunostaining of skin sections from 16 week-old mice with anti-FASN shows no difference in expression between wild-type and *Acer1^–/–^* mice, whereas ectopic expression of PPARγ is evident in *Acer1^–/–^* compared to wild-type epidermis (*n =* 3/age and genotype); scale bar = 100 μm

**Figure S7.** Normal epidermal barrier in *Acer1^–/–^* embryos. Barrier-dependent toluidine blue dye exclusion assay on E16.5–18.5 *Acer1* embryos shows that the dye fails to penetrate E17.5 embryos, indicating the formation of a fully functional skin barrier

**Figure S8.** Characterization of the hypermetabolic phenotype in *Acer1*^–/–^ mice. (A) Respiratory exchange ratio (RER) and (B) total spontaneous activity were measured using indirect calorimetry for 22 h (*n =* 27 *Acer1*^+/+^, *n =* 7 *Acer1*^–/–^ males). (C–F) Mice aged 9 and 32 weeks were weighed and culled to dissect out adipose tissue depots (*n =* 5/time point and genotype). (G) Quantitation of total UCP1 content/intrascapular BAT depot and (H) western blot images of BATs from 9 and 32 week-old wild-type and *Acer1*^–/–^ mice (*n =* 5/time point and genotype). Vinculin was used as the loading control; data are shown as mean ± SD and were analysed within each time point using Mann–Whitney test; ns, not significant; ***p <* 0.01
