# Supplementary figures and images for: Alkaline ceramidase 1 is essential for mammalian skin homeostasis and regulating whole‐body energy expenditure
Source: J Pathol. 2016 May 30;239(3):374–83. doi: 10.1002/path.4737 (PMC4924601; doi:10.1002/path.4737)

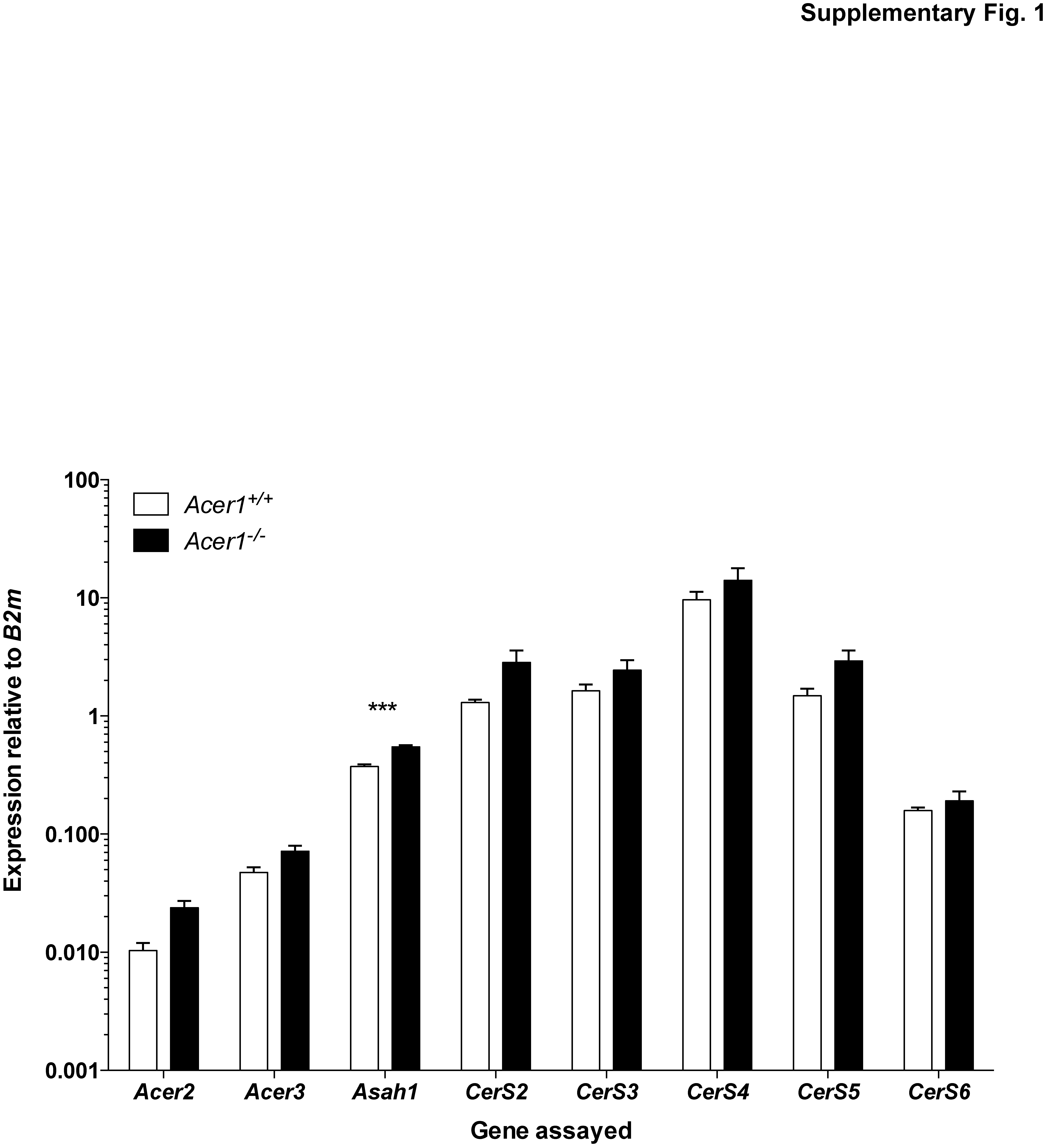

Supplement: Supplementary file 2 — RT–qPCR analysis of the levels of the ceramidases and ceramide synthases expressed in the skin of wild‐type and Acer1–/– mice relative to the endogenous control B2m (n = 4 Acer1 +/+, n = 3 Acer1 –/– females at age 7 weeks); Asah2 and CerS1 are not shown, as they are not expressed in mouse skin. Data are shown as mean ± SE/genotype; statistical analysis was by unpaired t‐test with adjustment for multiple testing for the individual enzymes species, using the Holm–Sidak method with α set to 5%; *** p = 0.0004 [file PATH-239-374-s007.tif]

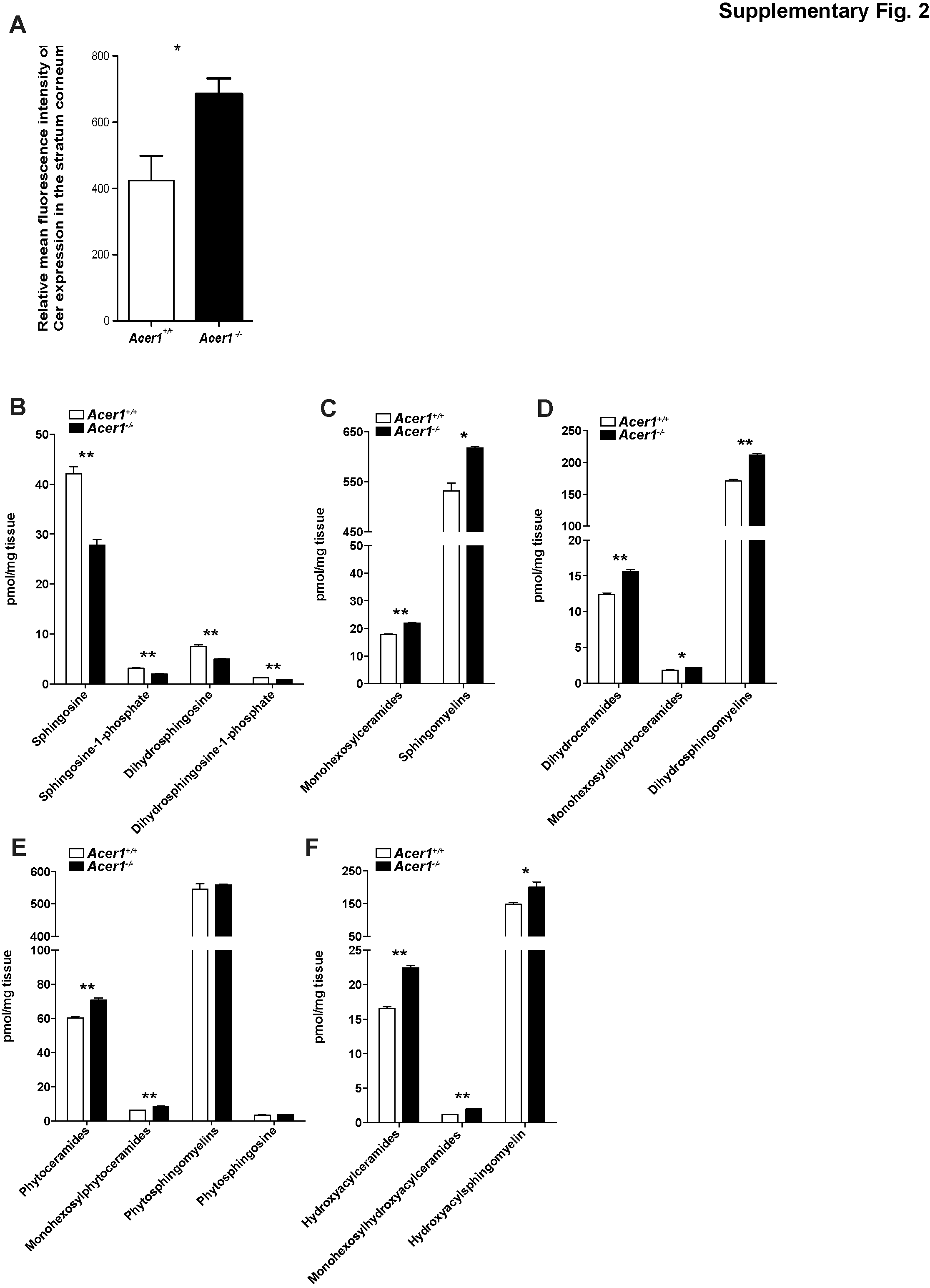

Supplement: Supplementary file 3 — Altered lipid composition of skin from Acer1–/– mice. (A) Quantification of ceramide content in the stratum corneum (SC) from 28 week‐old Acer1–/– mice shows increased total mean fluorescence intensity; data are mean ± SE (n = 2 males/genotype with two technical replicates); statistical analysis was by unpaired t‐test; *p = 0.0263. (B) Sphingosine, sphingosine‐1‐phosphate, dihydrosphingosine and dihydrosphingosine‐1‐phosphate. (C) Monohexosylceramides and sphingomyelins. (D) Dihydroceramides, monohexosyldihydroceramides and dihydrosphingomyelin. (E) Phytoceramides, hexosylphytoceramides, phytosphingomyelin and phytosphingosine. (F) Hydroxyacylceramides (which includes both 2‐hydroxy and omega‐O‐acylceramide), monohexosylhydroxyacylceramides and hydroxyacylsphingomyelin; data are mean ± SE (five males, aged 9 weeks)/tissue/genotype; statistical analysis was by unpaired t‐test with adjustment for multiple testing for the individual ceramide species, using the Holm–Sidak method with α set to 5%; *p < 0.05, **p < 0.01 [file PATH-239-374-s005.tif]

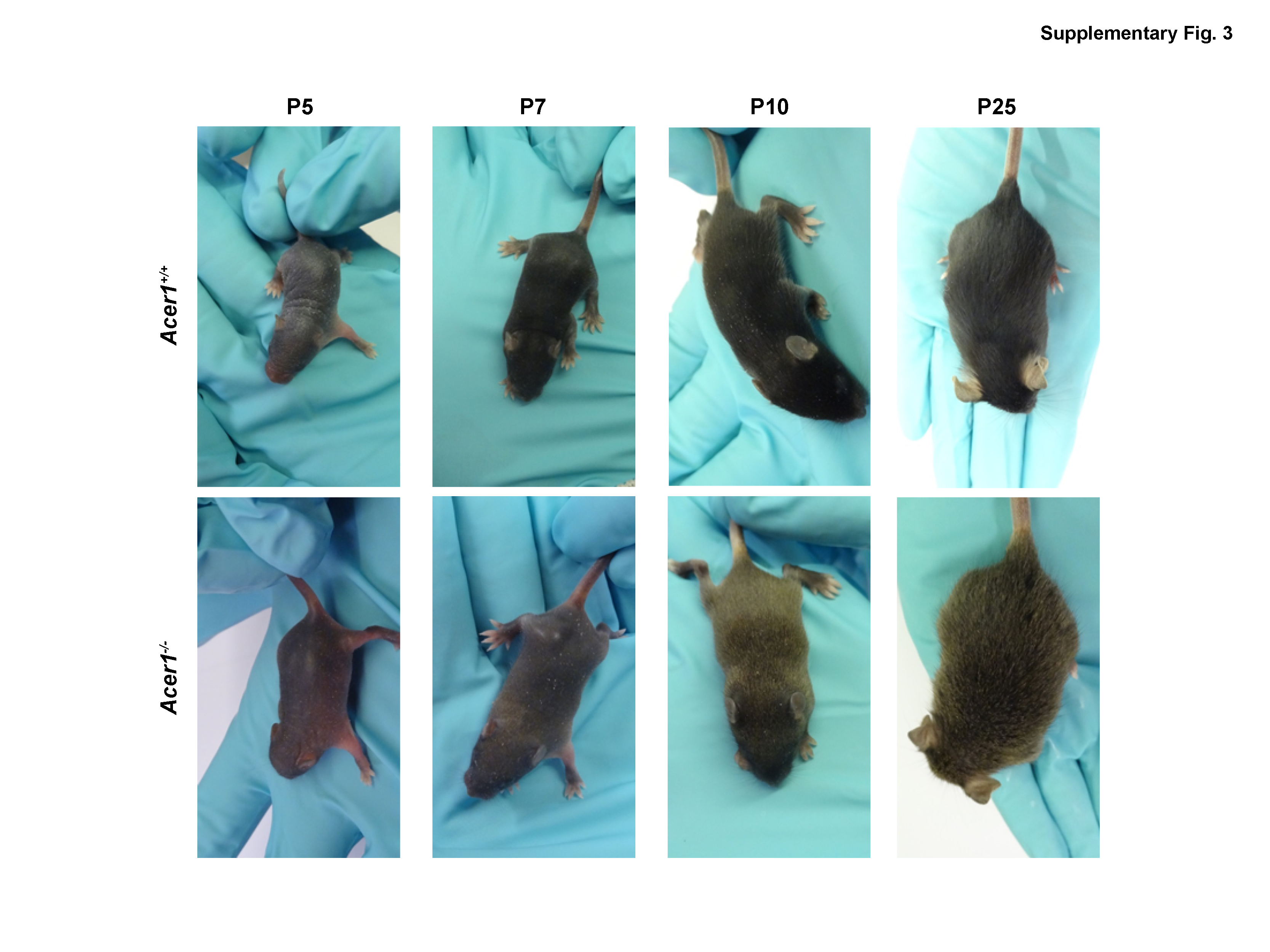

Supplement: Supplementary file 4 — Acer1 –/– pups have normal onset of hair growth. Dorsal images of Acer1 +/+ and Acer1 –/– male pups taken 5–25 days postpartum, demonstrating the similarity in the timing of hair growth between genotypes. By P25, Acer1 –/– male display the abnormal hair length phenotype [file PATH-239-374-s003.tif]

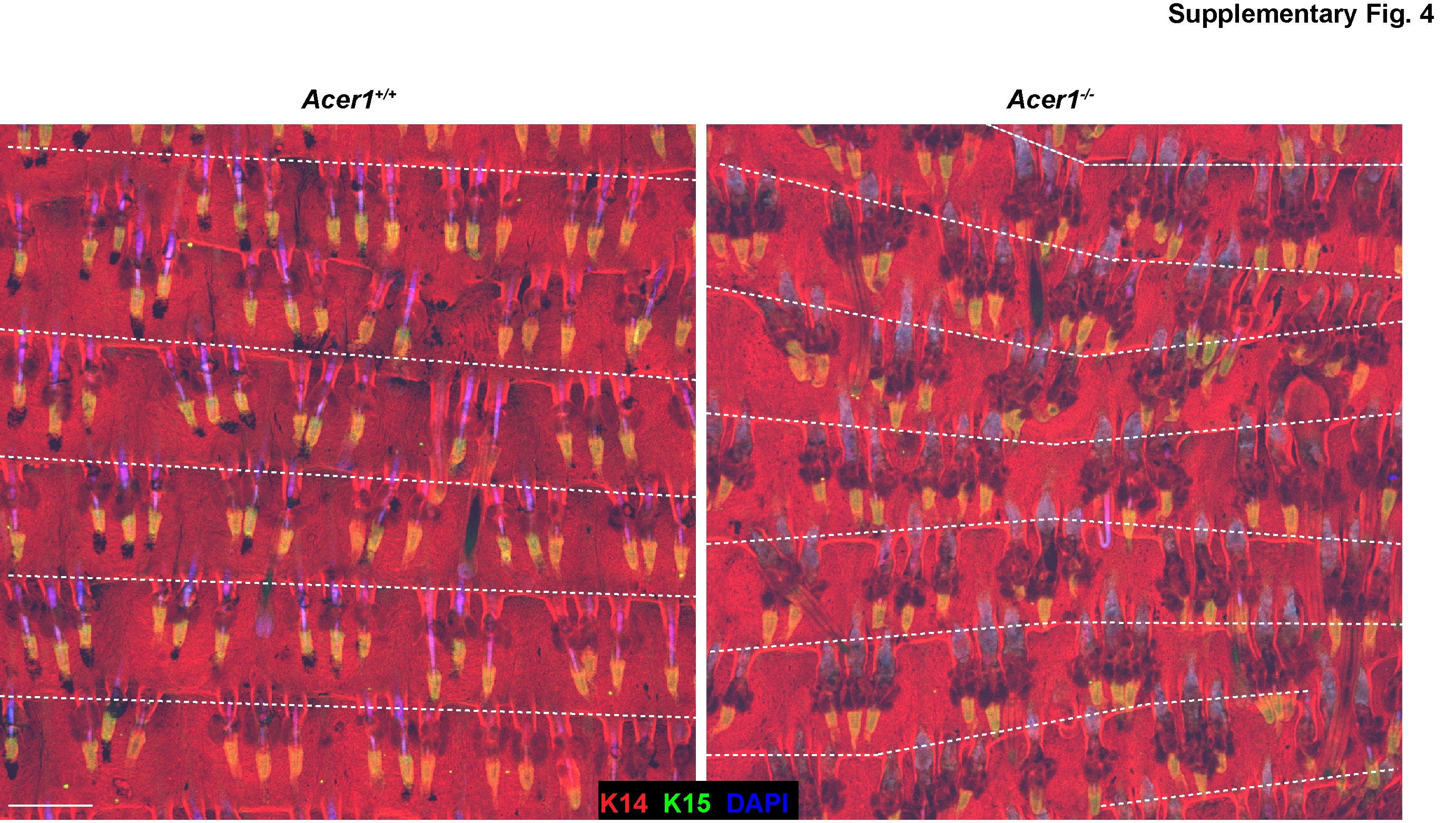

Supplement: Supplementary file 5 — Altered hair follicle patterning in Acer1–/– epidermis. Representative images of tail epidermal whole mount with K14 and K15 staining, showing irregular arrangement of hair follicle triplet clusters in Acer1–/– epidermis when compared to wild‐type (n = 3/age and genotype); scale bar = 100 µm [file PATH-239-374-s008.tif]

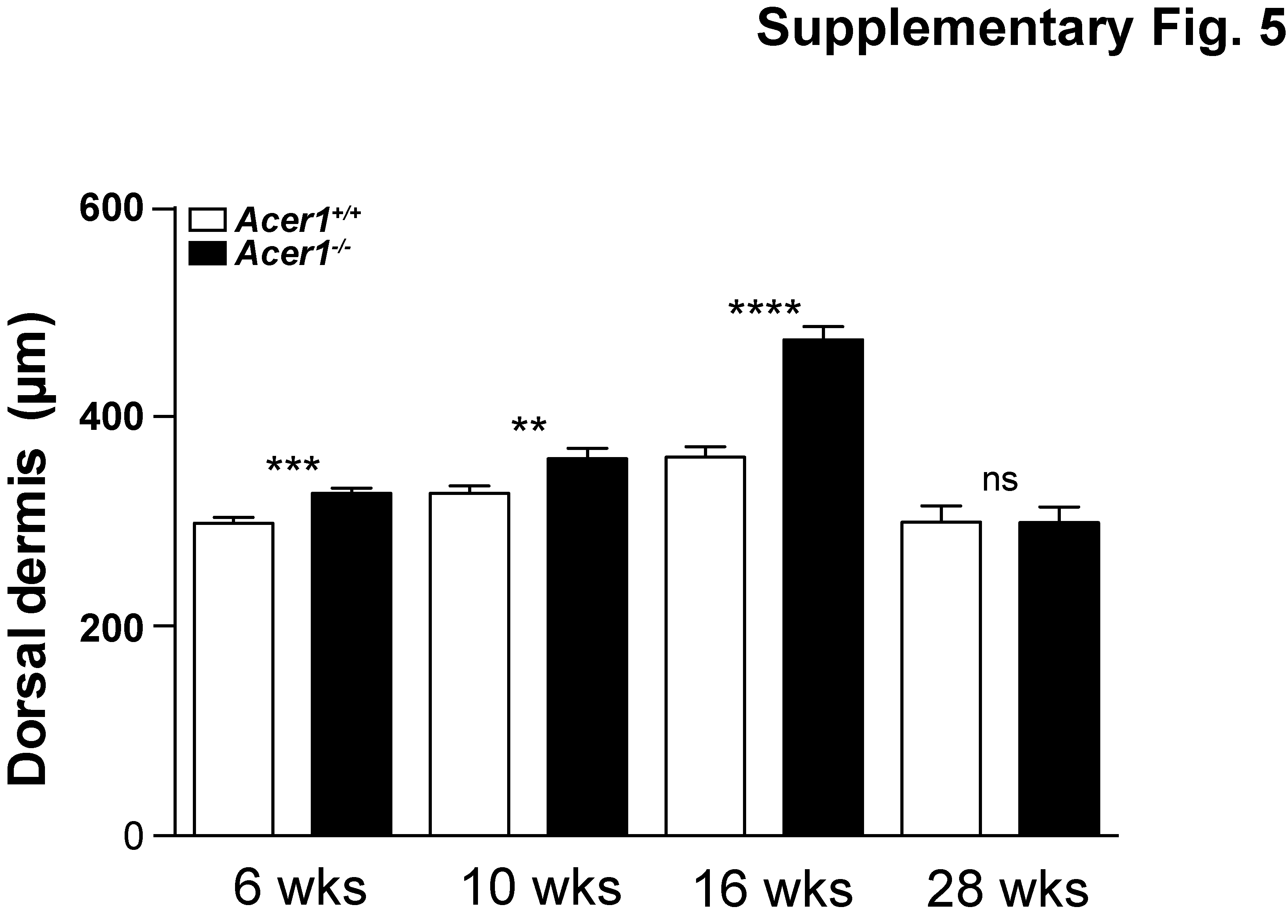

Supplement: Supplementary file 6 — Acer1–/– mice have an altered skin phenotype. Quantification of dorsal dermis thickness at different ages; data are shown as mean ± SD (n = 3) and analysed using unpaired t‐test; ns, not significant; **p = 0.0013, ***p = 0.0010, ****p < 0.0001 [file PATH-239-374-s009.tif]

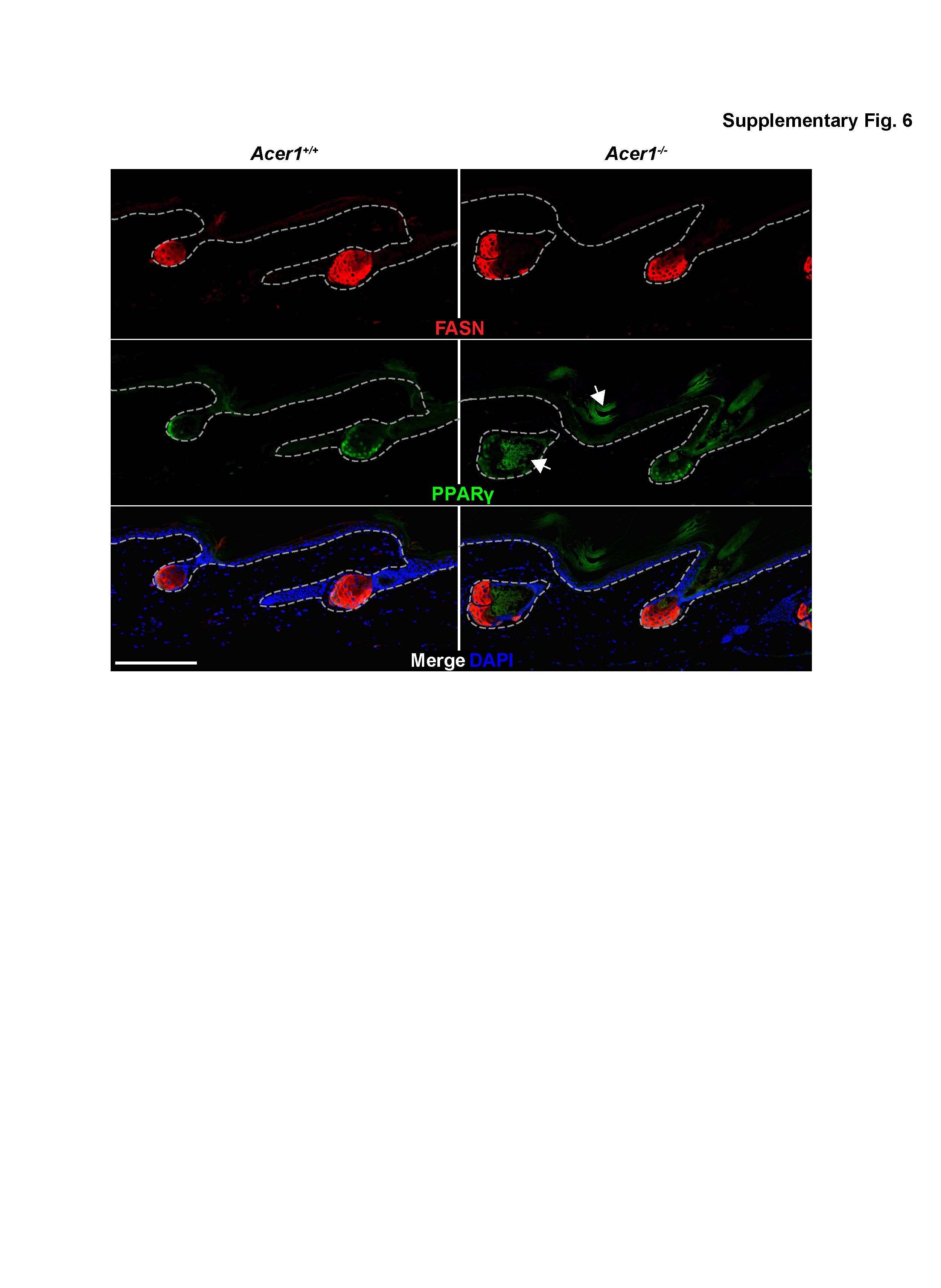

Supplement: Supplementary file 7 — Expression of sebaceous gland differentiation markers in Acer1 epidermis. Representative immunostaining of skin sections from 16 week‐old mice with anti‐FASN shows no difference in expression between wild‐type and Acer1–/– mice, whereas ectopic expression of PPARγ is evident in Acer1–/– compared to wild‐type epidermis (n = 3/age and genotype); scale bar = 100 µm [file PATH-239-374-s006.tif]

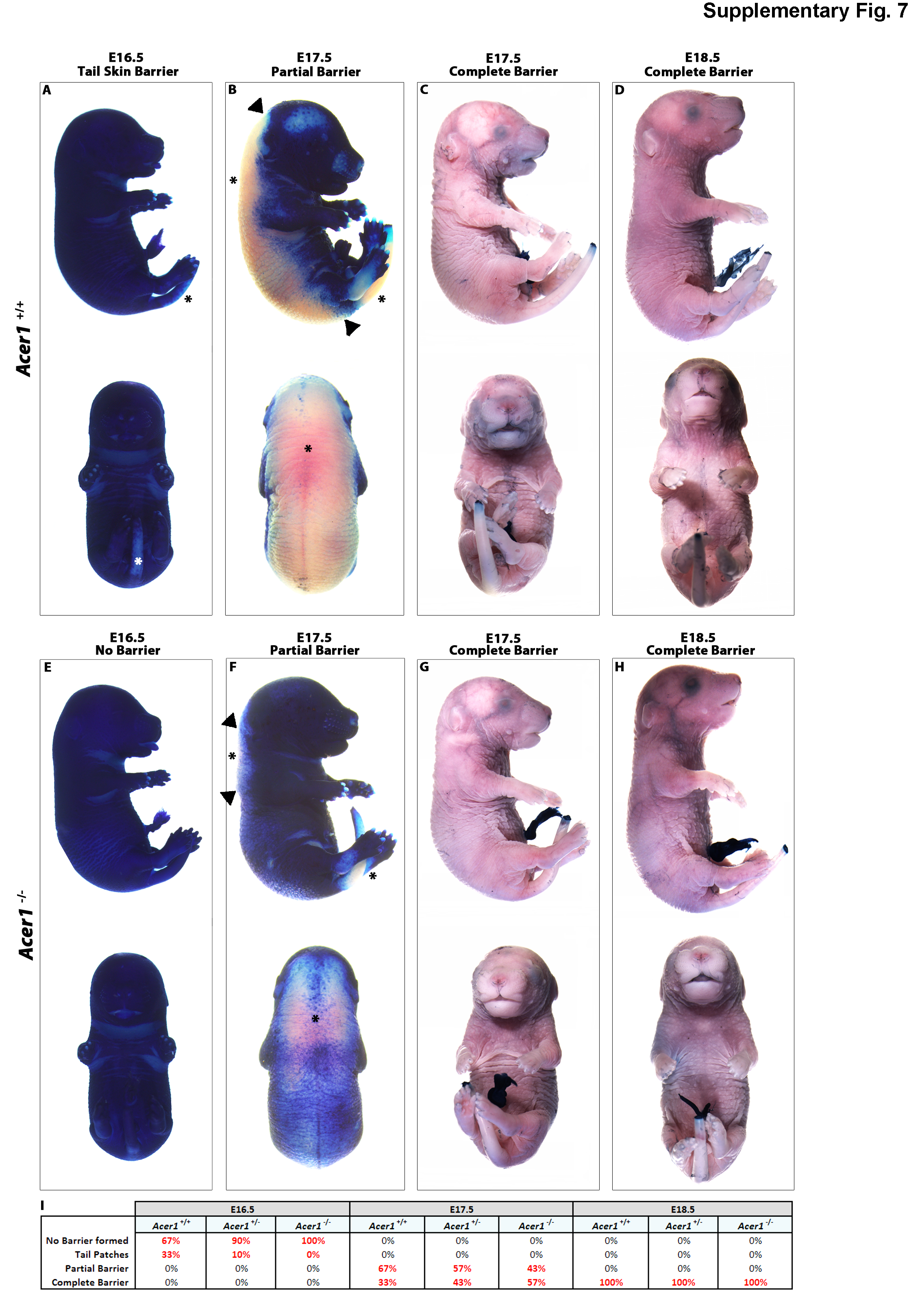

Supplement: Supplementary file 8 — Normal epidermal barrier in Acer1–/– embryos. Barrier‐dependent toluidine blue dye exclusion assay on E16.5–18.5 Acer1 embryos shows that the dye fails to penetrate E17.5 embryos, indicating the formation of a fully functional skin barrier [file PATH-239-374-s004.tif]

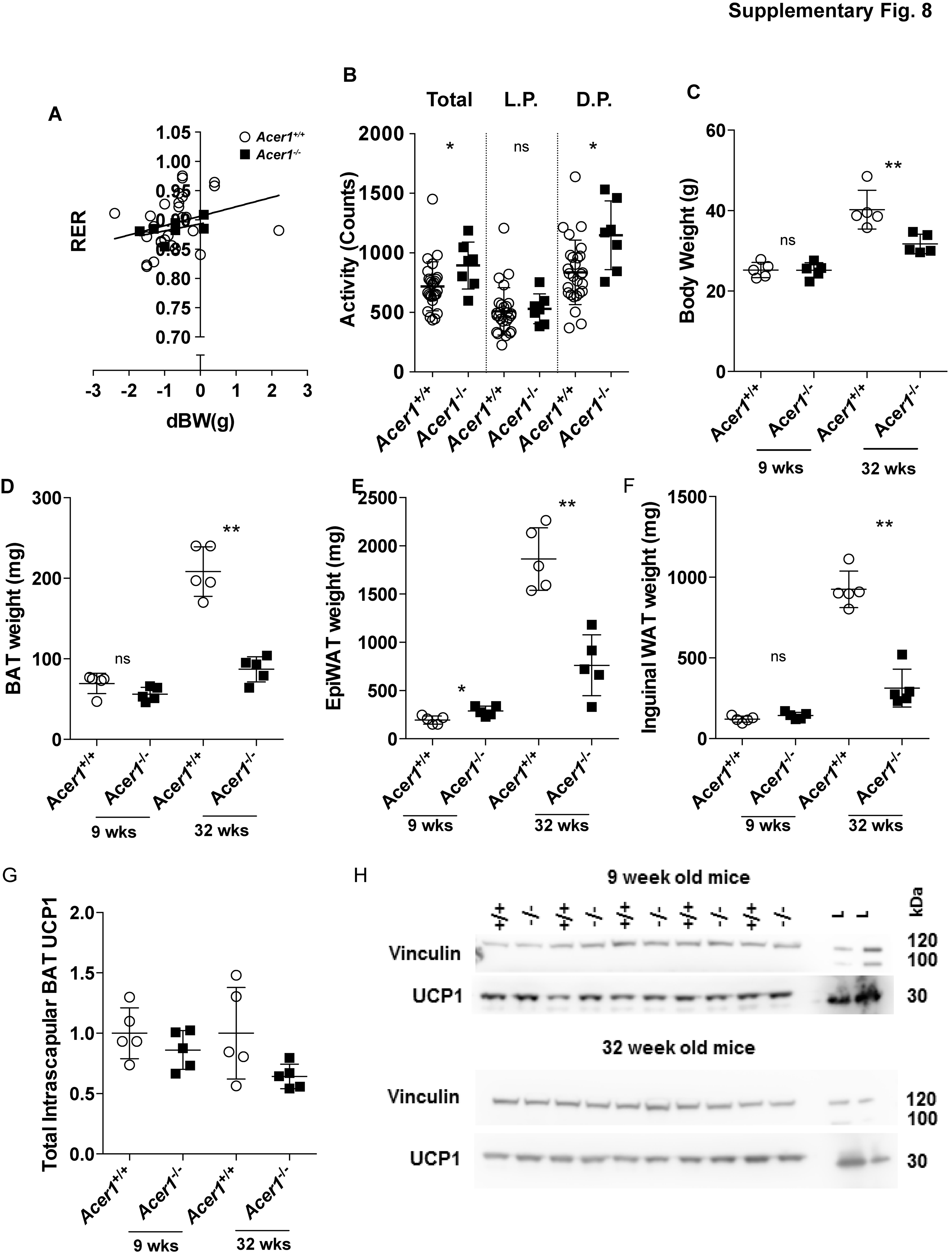

Supplement: Supplementary file 9 — Characterization of the hypermetabolic phenotype in Acer1 –/– mice. (A) Respiratory exchange ratio (RER) and (B) total spontaneous activity were measured using indirect calorimetry for 22 h (n = 27 Acer1 +/+, n = 7 Acer1 –/– males). (C–F) Mice aged 9 and 32 weeks were weighed and culled to dissect out adipose tissue depots (n = 5/time point and genotype). (G) Quantitation of total UCP1 content/intrascapular BAT depot and (H) western blot images of BATs from 9 and 32 week‐old wild‐type and Acer1 –/– mice (n = 5/time point and genotype). Vinculin was used as the loading control; data are shown as mean ± SD and were analysed within each time point using Mann–Whitney test; ns, not significant; **p < 0.01 [file PATH-239-374-s010.tif]
