# Supplementary material for: Alkaline ceramidase 1 is essential for mammalian skin homeostasis and regulating whole‐body energy expenditure
Source: J Pathol. 2016 May 30;239(3):374–83. doi: 10.1002/path.4737 (PMC4924601; doi:10.1002/path.4737)
Supplement: Supplementary file 11 — Dysmorphology analysis of 10 week‐old Acer1 –/– and wild‐type mice [file PATH-239-374-s012.docx]

**Table S2**. Dysmorphology analysis of 10 week-old *Acer1*^–/–^ and wild-type mice

|  |  | Male Acer1^+/+^ | Female Acer1^+/+^ | Male Acer1^–/–^ | Female Acer1^–/–^ | Genotype effect |
| --- | --- | --- | --- | --- | --- | --- |
| Head coat/hair presence | As expected | 8 | 8 | 0 | 4 | 0.0001 |
|  | Patchy | 0 | 1 | 0 | 0 |  |
|  | Patchy and sparse | 0 | 0 | 7 | 3 |  |
|  | Sparse | 0 | 0 | 0 | 1 |  |
| Head coat/hair length | As expected | 8 | 9 | 0 | 0 | < 0.001 |
|  | Mixed | 0 | 0 | 7 | 8 |  |
| Head skin appearance | As expected | 8 | 9 | 0 | 8 | 0.0013^#^ |
|  | Dry | 0 | 0 | 7 | 0 |  |
|  | Scaly | 0 | 0 | 0 | 0 |  |
| Dorsal coat/hair presence | As expected | 8 | 9 | 0 | 1 | < 0.001 |
|  | Patchy | 0 | 0 | 0 | 0 |  |
|  | Patchy and sparse | 0 | 0 | 5 | 5 |  |
|  | Sparse | 0 | 0 | 2 | 2 |  |
| Dorsal coat/hair length | As expected | 8 | 9 | 0 | 0 | < 0.001 |
|  | Mixed | 0 | 0 | 7 | 8 |  |
| Dorsal skin appearance | As expected | 8 | 9 | 7 | 7 | NS |
|  | Dry | 0 | 0 | 0 | 1 |  |
|  | Scaly | 0 | 0 | 0 | 0 |  |
| Ventral coat/hair presence | As expected | 8 | 9 | 0 | 2 | < 0.001 |
|  | Patchy | 0 | 0 | 0 | 0 |  |
|  | Patchy and sparse | 0 | 0 | 1 | 0 |  |
|  | Sparse | 0 | 0 | 6 | 6 |  |
| Ventral skin appearance | As expected | 8 | 9 | 0 | 6 | 0.0001 |
|  | Dry | 0 | 0 | 0 | 0 |  |
|  | Scaly | 0 | 0 | 7 | 2 |  |
| Limb coat/hair presence | As expected | 8 | 8 | 0 | 5 | 0.002^#^ |
|  | Patchy | 0 | 1 | 0 | 0 |  |
|  | Patchy and sparse | 0 | 0 | 5 | 3 |  |
|  | Sparse | 0 | 0 | 2 | 0 |  |
| Ventral skin appearance | As expected | 8 | 9 | 0 | 6 | 0.0001 |
|  | Dry | 0 | 0 | 1 | 0 |  |
|  | Scaly | 0 | 0 | 6 | 2 |  |

The data indicate the number of mice with the recorded phenotype (male *Acer1*^+/+^, *n =* 8; female *Acer1*^+/+^, *n =* 9; male *Acer1*^–/–^, *n =* 7; male *Acer1*^–/–^, *n =* 8). Data are analysed with logistic regression to give the *p* value for genotype effects, with all phenotypes other than 'as expected' classed as abnormal for the purposes of analysis.

NS, not significant.

^#^Significance relates only to males.
